# Assessing the climate change impact on *Epimedium brevicornu* in China with the MaxEnt model

**DOI:** 10.3389/fpls.2025.1534608

**Published:** 2025-06-16

**Authors:** Quanwei Liu, Zhihang Zhuo, Zhiling Wang, Yaqin Peng, Danping Xu

**Affiliations:** College of Life Science, China West Normal University, Nanchong, China

**Keywords:** *Epimedium brevicornu*, MaxEnt, climate change, potential suitable distribution, environmental variables

## Abstract

*Epimedium brevicornu* is a traditional medicinal plant in China, containing rich and medically valuable extracts. In recent years, the widespread development and application of its extracts have threatened the wild population of *E.brevicornu*. In order to protect the population of *E. brevicornu*, this research employed the Maxent model to examine the influence of climate change on the geographical distribution of *E. brevicornu* and to forecast its potential suitable distribution in China in light of climate change scenarios. The suitable habitat for *E. brevicornu* is located between 25.13°-39.50°N and 102.46°-118.13°E, mainly distributed across Loess Plateau. Climate change has a significant impact on the geographic distribution of *E. brevicornu*, with its high suitability zone expected to increase in the future and its centroid shifts towards the southeast direction. The 2050s projections under the Shared Socioeconomic Pathways (SSP) 1-2.6 and SSP2-4.5 scenarios indicated a significant expansion of highly suitable habitats. The analysis of key environmental variables showed that the seasonal variation coefficient of temperature (bio4), the lowest temperature in the coldest month (bio6), annual precipitation (bio12), seasonal variation of precipitation (bio15), human activity (hf), and the average ultraviolet radiation (UV-B3) in the highest month were the key factors affecting *E. brevicornu* selection of suitable habitats. This study provided important reference for the protection of the wild population of *E. brevicornu* and the selection of artificial planting areas in the future.

## 1 Introduction

Medicinal plants are called traditional Chinese medicine in China, referring to plants with medicinal value. They can be used either in a traditional way of water decoction, or extract their active ingredients with medicinal value through modern scientific and technological way to make medicine (Cheng et al., 2021). Medicinal plants have a long history in relieving pain and treating diseases around the world (Mulugeta et al., 2023). In the research of the new era, medicinal plants are an important source of abundant medical extracts, promoting the birth of many modern drugs. Over the past 20 years, more than two-thirds of newly launched small molecule drugs have come from plant extracts (Rai et al., 2017). In addition, plant extracts also have great potential in antibacterial research (Mulugeta et al., 2023). *Epimedium brevicornu* belongs to the family Berberidaceae and the genus *Epimedium*, which is one of the largest genera of perennial herbaceous plants in the world (Li et al., 2024a), with about 68 species under its genus, including over 50 in China (Zhang et al., 2023). As a medicinal plant, it has important medicinal value in its leaves (Xue et al., 2023). Due to the relative stability of its medicinal components, this plant increasingly attracts the attention of pharmacognologists (Li et al., 2010). Previous studies had shown that *E. brevicornu* was rich in various bioactive components, including but not limited to flavonoids, lignin, organic acids, terpenoids, dihydrophenanthrene derivatives, amino acids, and alkaloids (Xue et al., 2023; Zhang et al., 2023; Qian et al., 2024). Flavonoids in this species had been identified as the main active substances with pharmacological effects (Lin et al., 2023). *E. brevicornu* has been proven to have good therapeutic effects on symptoms such as osteoporosis and kidney yang deficiency (Liu et al., 2018; Wang et al., 2023a). As a traditional Chinese medicine, *E. brevicornu* could also treat rheumatism, hypertension, and coronary heart disease, enhance immunity, and prevent dementia (Zhang et al., 2018a). *Epimedium* plants have enormous commercial value, but with the development and application of modern extraction technology, the global use of medicinal plants are increasing rapidly, and wild *Epimedium* populations are facing threats (Zaiyou et al., 2022). Therefore, population protection has become crucial.

*Epimedium brevicornum* is native to China and is mainly distributed in Shaanxi, Guizhou, Shanxi, Henan, Qinghai, Hubei, and Sichuan (Ma et al., 2011). The relationship between the geographical distribution of the research species and environmental factors is an important topic in ecological research (Lawler et al., 2009). Studies have shown that the potential distribution of a species can be inferred by studying the climate of a species’ range (Wang et al., 2021). Species Distribution Models (SDMs) are a method to estimate the geographical distribution of species based on their actual distribution (Yang et al., 2022). They are widely used in fields such as conservation biology and ecology (Gobeyn et al., 2019; Hao et al., 2019), also in studying the historical geographical distribution of species and their distribution trends under future climate change (Zhang and Wang, 2023). The MaxEnt model, proposed by Phillips in 2004, is currently one of the most popular methods for establishing species distribution models (Phillips et al., 2004; Dai et al., 2022). It uses the maximum entropy principle and species records to predict the potential distribution of species (Phillips et al., 2004). The MaxEnt models has the advantages of short running time, ease of operation, good performance, and high accuracy (Warren and Seifert, 2011; He et al., 2021). The optimized MaxEnt model exhibits higher stability compared to other models (Zhang and Wang, 2022) and has demonstrated greater accuracy than some ensemble models in certain cases (Yu and Li, 2024; Lv et al., 2025). This model determined the range of possibilities through 10 experiments and created a distribution model based on the average output of these experiments (Phillips et al., 2006). MaxEnt modeling had been widely used in the fields of species conservation and crop regionalization (Cao et al., 2016; Kaky et al., 2020; Wang et al., 2020). Previously, the model had been applied to the potential distribution research of various plants such as *Ephedra sinica*, *E. intermedia* and *E. equisetina* (He et al., 2021), *Polygonatum kingianum* (Guo et al., 2023), *Houttuynia cordata* (Liu et al., 2021), and *Leonurus japonicus* (Wang et al., 2023b). These studies have provided valuable references for the development and utilization of medicinal plant resources.

Currently, researches on *E. brevicornu* mainly focus on the study of its active substances, and few studies have been done to predict its distribution. In order to protect the wild populations of *E. brevicornu*, it is necessary to predict its potential distribution. This study utilized the simple and efficient MaxEnt model to identify suitable habitats for *E. brevicornum* in the region. The relationship between environmental variables and *E. brevicornum* was explored, and the influence of various environmental parameters on its distribution was assessed. In order to effectively protect the resources of *E. brevicornu*, the MaxEnt model was also used to simulate suitable habitats and comprehensively analyze all results to select the priority protection areas for *E. brevicornu*. Revealed the main limiting factors of the habitat and future distribution of *E.brevicornu*, determined its suitable planting area, these findings provide a scientific reference provide reference for its planting and protection.

## 2 Materials and methods

### 2.1 Species occurrence records

Since the first proposal of the MaxEnt model in 2004, researchers have clearly emphasized that high-quality species occurrence data were needed for high-quality statistical analysis (Phillips et al., 2004). Studies have shown that MaxEnt performs well for species with few occurrence records and is more suitable for predicting the distribution of rare species (Jha et al., 2022). Therefore, MaxEnt was selected in this study to model and predict the future distribution of *E. brevicornum*. Species distribution data were primarily obtained from the Global Biodiversity Information Facility (GBIF.org (8 July 2024) GBIF Occurrence Download https://doi.org/10.15468/dl.2ktupd), Chinese Virtual Herbarium (http://www.cvh.ac.cn), and keyword literature searches. The longitude and latitude distribution data of this species were determined by using Google Maps (http://ditu.google.cn/). In order to ensure data quality and reduce the impact of data duplication and redundancy on the results, duplicate points within each grid cell (10 km × 10 km) were removed (Zhang et al., 2018b), resulting in a total of 92 distribution points.

### 2.2 Environment variables

The model first selected 19 bioclimatic variables from the WorldClim global climate database (https://worldclim.org/) (**Supplementary Table S1**). To eliminate multicollinearity among these variables, MaxEnt v3.4.1 was initially used to calculate the contribution rates of the 19 environmental variables (**Supplementary Table S2**). Then, Pearson correlation analysis was conducted using SPSS 25.0 to exclude environmental factors with an absolute correlation coefficient greater than or equal to 0.8 (Feng et al., 2019; Jayasinghe and Kumar, 2019), resulting in the selection of six bioclimatic variables (**Supplementary Table S3**). Since various soil components and microorganisms can influence plant growth and distribution, incorporating soil and topographic factors into the species distribution model was necessary (Jiawen et al., 2022; Shen et al., 2024). Therefore, topographic variables, including elevation (alt), slope, and aspect, were obtained from the Resource and Environmental Science and Data Center of the Institute of Geographic Sciences and Natural Resources Research, Chinese Academy of Sciences (https://www.resdc.cn/). Soil variables, including soil pH, topsoil sand content (T-sand), topsoil organic carbon content (T-C), reference soil depth (ref), and soil texture classification (t-usda-te), were sourced from the World Soil Database (https://gaez.fao.org/pages/hwsd). Additionally, the highest monthly mean UV-B radiation (UV-B3) was retrieved from the gIUV database (http://www.ufz.de/gluv/), and the human footprint index (hf) was acquired from the Center for International Earth Science Information Network (CIESIN) (http://www.ciesin.org/). To comprehensively investigate the effects of soil and topographic factors on *E. brevicornu*, these variables, along with the six previously selected bioclimatic factors, were all incorporated into MaxEnt v3.4.1 for final modeling (**Table 1**). All environmental factor data were from the 2023 release.

**Table 1:** Environmental variables affecting *E. brevicornu* distribution.

Code	Variable	Unit
bio3	Isothermality	%
bio4	Temperature Seasonality	°C
bio6	Min Temperature of Coldest Month	°C
bio8	Mean Temperature of Wettest Quarter	°C
bio12	Annual Precipitation	mm
bio15	Precipitation Seasonality	mm
alt	Altitude	m
aspect	aspect	°
slope	slope	°
PH	Potential of hydrogen	/
t-sand	Topsoil sand fraction	%
t-C	Topsoil organic carbon	%
ref-depth	Reference soil depth	m
t-usda-te	USDA Soil texture classification	/
UV-B3	Mean UV-B of Highest Month	kJ/m^2^
hf	Human footprint index	/

The model was developed based on the Shared Socioeconomic Pathways (SSP) of the Coupled Model Intercomparison Project Phase 6 (CMIP6), proposed by the Intergovernmental Panel on Climate Change (IPCC) (Eyring et al., 2016). Following previous studies, three commonly used SSP scenarios—SSP1-2.6, SSP3-7.0, and SSP5-8.5—were selected for modeling from a total of 23 scenarios (Xu et al., 2024).

### 2.3 Modeling process and optimization

During the modeling process, 25% of the data were randomly selected for testing, while the remaining 75% were used as the training set. This procedure was repeated ten times to establish the predictive model. Subsequently, the “kuenm” package in R was used to optimize the regularization multiplier (RM) and feature combination (FC) parameters (Zeng et al., 2016; Kass et al., 2021). The specific procedure was as follows: First, five feature combinations (FCs) and their corresponding regularization multipliers (RMs) were set, generating five candidate models. Then, the “kuenm” package was used to evaluate these models based on the selected parameter combinations. The optimal model was chosen from the candidate models based on two criteria: an omission rate (OR) of <5% and a Delta AICc (Akaike Information Criterion, AIC) value of <2. Finally, among the optimal models, the one with the smallest Delta AICc value was selected as the optimized model, determining its regularization multiplier and feature combination parameters.

### 2.4 Model evaluation

The prediction performance indicators of the MaxEnt model include the area under the ROC curve (AUC), the Kappa coefficient (KAPPA), and the True Skill Statistic (TSS). The model’s predictive accuracy is directly proportional to these evaluation metrics. When the value falls between 0.7 and 0.85, it indicates that the model performs well; a value between 0.85 and 1 suggests that the model has excellent performance (Yang et al., 2022; Zhang et al., 2024a).

The distribution data generated by MaxEnt within China was extracted using ArcGIS 10.8 software, and the climate suitability of *E. brevicornu* was studied in depth based on the existence probability generated by MaxEnt. The suitability was classified into four levels based on the existence probability: highly suitable (0.6-1), suitable (0.3-0.6), less suitable (0.1-0.3), and unsuitable (0-0.1) (Zhang et al., 2018b).

## 3 Results

### 3.1 Model performance and variable selection

**Figure 1:**
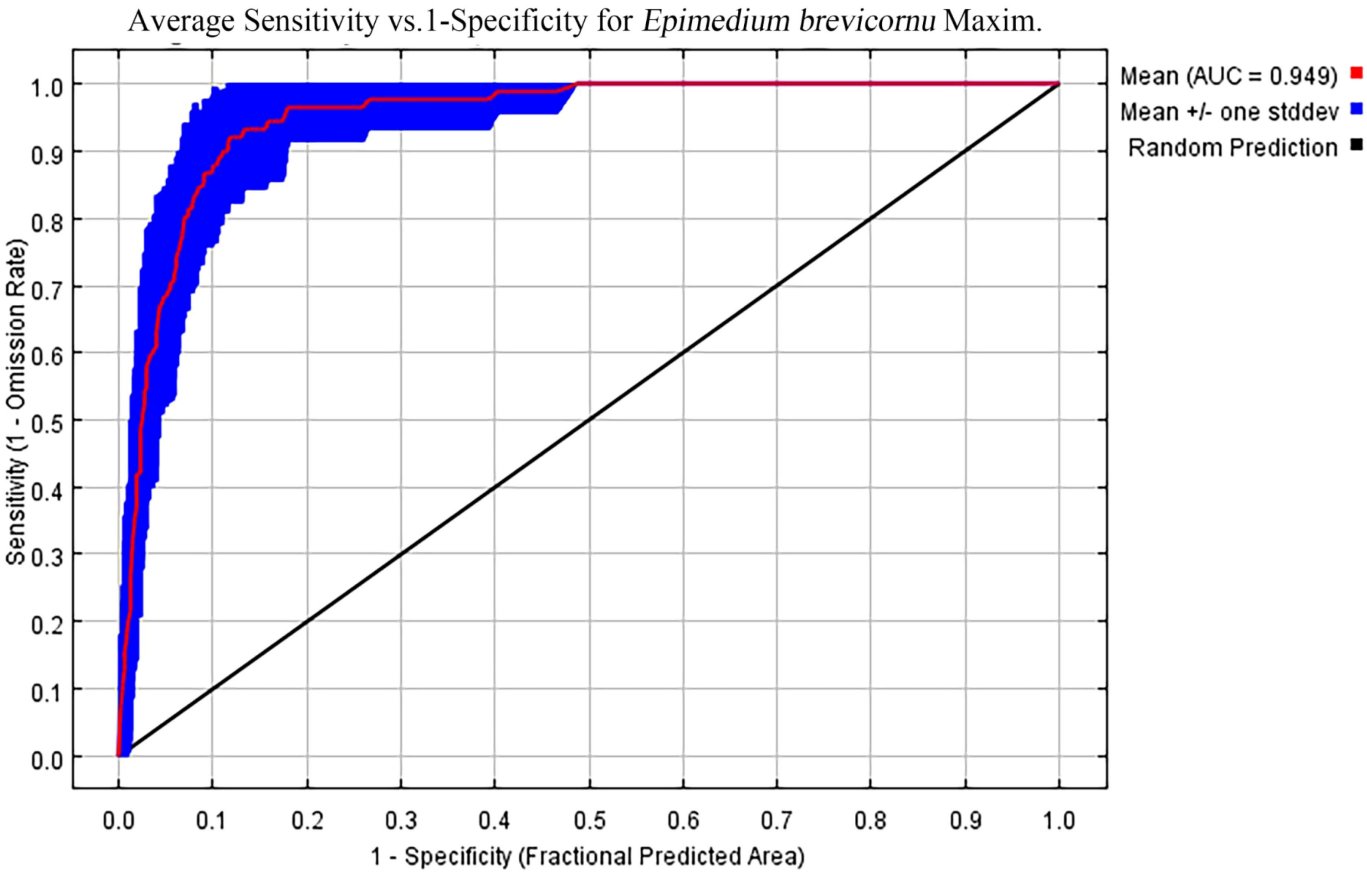
The receiver operating characteristic (ROC) curve and area under the curve (AUC) values of the MaxEnt model for *E. brevicornu*.

**Figure 2:**
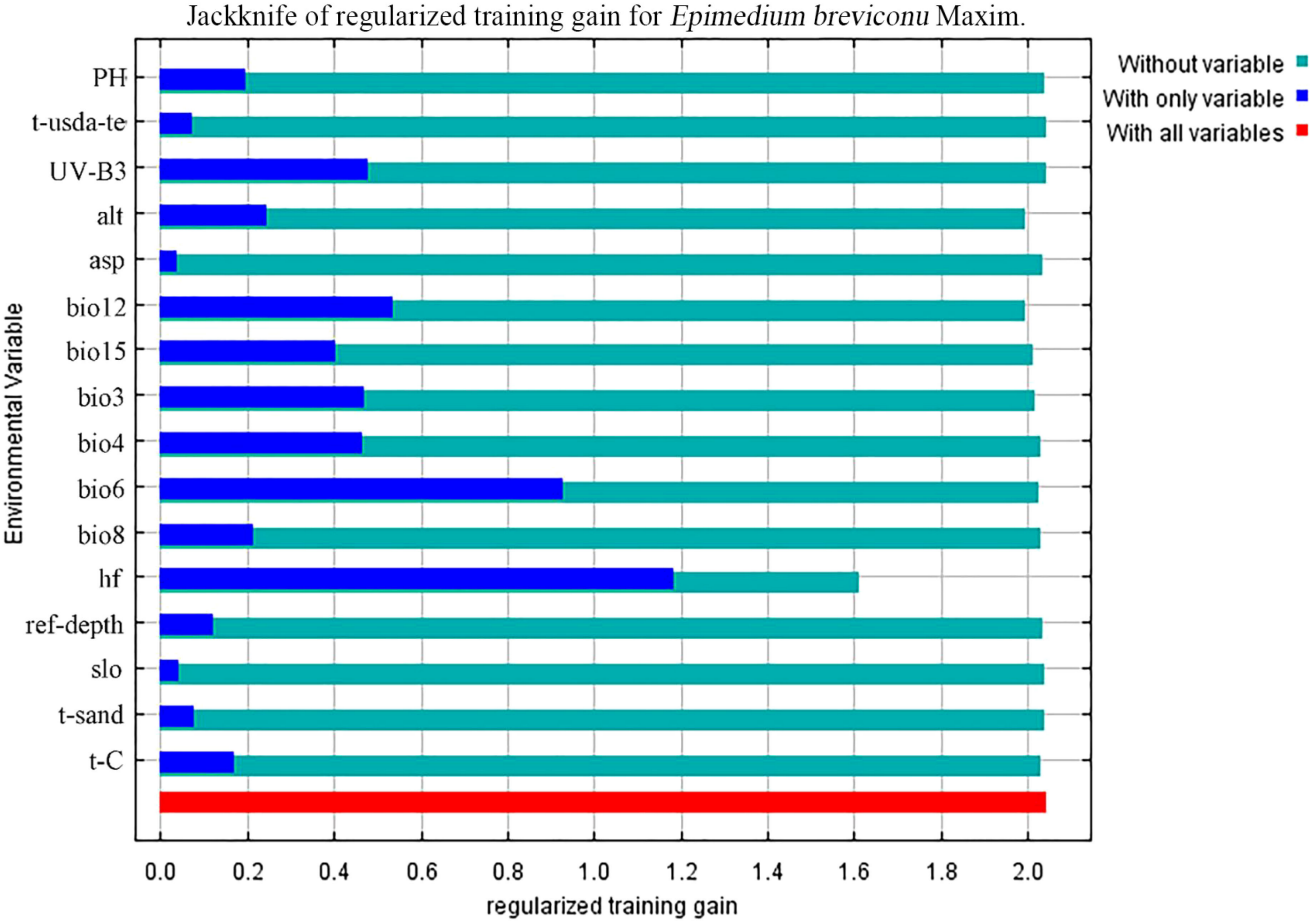
Assessing the importance of environmental variables affecting the distribution of *E. brevicornu* using the Jackknife test. The blue bars indicate the importance of each environmental variable.

In the ten trials, the AUC value of the *E. brevicornum* distribution model was 0.949 (**Figure 1**), the TSS value was 0.860, and the KAPPA value was 0.786 (**Table 2**). Although the TSS and KAPPA values are lower than the AUC, they still indicate that the model provides good prediction accuracy for the distribution of *E. brevicornum*. After screening, 6 out of 19 bioclimatic variables were included in the modeling, with a cumulative contribution rate of 74.7%, namely: minimum temperature of the coldest month (bio6) (29.1%), annual precipitation (bio12) (17.2%), precipitation seasonality (bio15) (14.3%), temperature seasonality (bio4) (9.4%), isothermality (bio3) (3.2%), and mean temperature of the wettest quarter (bio8) (1.5%). Because the model needed to consider factors with relatively high contribution rates (Wei et al., 2023), bio3 and bio8, with low contribution rates, were not considered key environmental factors in this work. In the final model, based on the training gains of different climatic factors analyzed using the jackknife method, the top 4 climatic factors in terms of training gain scores were human footprint (hf), bio6, bio12, and Mean UV-B of Highest Month (UV-B3) (**Figure 2**). Considering both the contribution rate ranking and the training gain ranking, the above-mentioned 6 environmental factors (bio6, bio4, bio12, bio15, hf, UV-B3) were analyzed as key environmental factors affecting the distribution of *E. brevicornu*.

**Table 2:** TSS and Kappa values for MaxEnt model performance.

Index name	Value
TSS	0.860
KAPPA	0.786

### 3.2 Potential distribution of *E. brevicornu* in the current period

**Figure 3:**
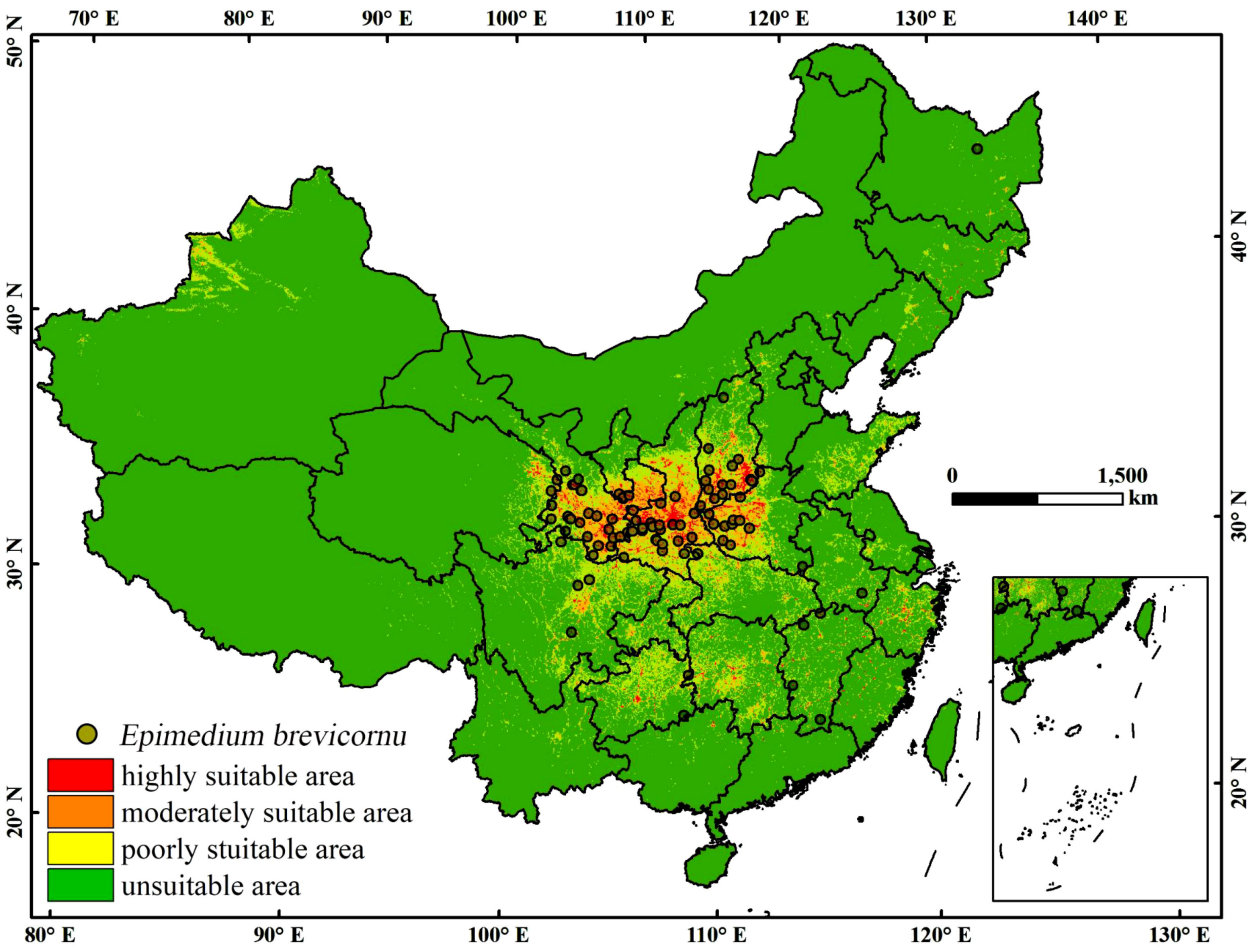
Current distribution of *E. brevicornu* under the present scenario. The circle in the graph represents the record of *E. brevicornu*’s accidents. This area’s color blocks display the probability of *E. brevicornu* occurrence. Red indicates a high suitability range of 0.6-1, orange indicates a moderate suitability range of 0.3-0.6, yellow indicates a low suitability range of 0.1-0.3, and green indicates an unsuitable range of 0-0.1.

The MaxEnt model was used to construct the most suitable habitat prediction map for *E. brevicornu* (**Figure 3**), The model’s predictions closely matched the current distribution of *E. brevicornum*. Under the current climatic conditions, the high suitability score zone for *E. brevicornu* was mainly concentrated in the area of 25.13°-39.50°N, 102.46°-118.13°E, primarily located in the Loess Plateau. In addition, certain suitable areas were also identified in the Sichuan Basin, Yunnan-Guizhou Plateau, and some parts of the southeastern coast.

According to the predicted area of *E. brevicornu* under the current climate conditions (**Supplementary Table S4**; **Table 3**), Gansu, Shanxi, Shaanxi, and Henan provinces had a relatively high proportion of high suitability areas, with their respective area proportions of 4.12%, 7.06%, 11.75%, and 4.84% (**Table 3**). The total areas of high, medium, and low suitable areas in these provinces was 6 × 10³ km², 19 × 10³ km², and 25 × 10³ km², respectively, with a total area of 44 × 10³ km² (**Table 3**).

**Table 3:** Core provinces and the proportion of the area covered by the current distribution of *E. brevicornum*.

Decade Scenarios	Predicted area (×10^3^ km^2^)			Area ratio (%)		
	Poorly suitable aera	Moderately suitable aera	Highly suitable aera	Poorly suitable aera	Moderately suitable aera	Highly suitable aera
Gansu	7.06	6.25	1.71	17.00	15.05	4.12
Shanxi	5.25	2.91	1.13	32.92	18.25	7.06
Shaanxi	7.91	6.48	2.39	38.79	31.79	11.75
Henan	4.75	3.35	0.78	29.44	20.80	4.84
Total	25	19	6			

### 3.3 Potential distribution of *E. brevicornu* in the future period

**Figure 4:**
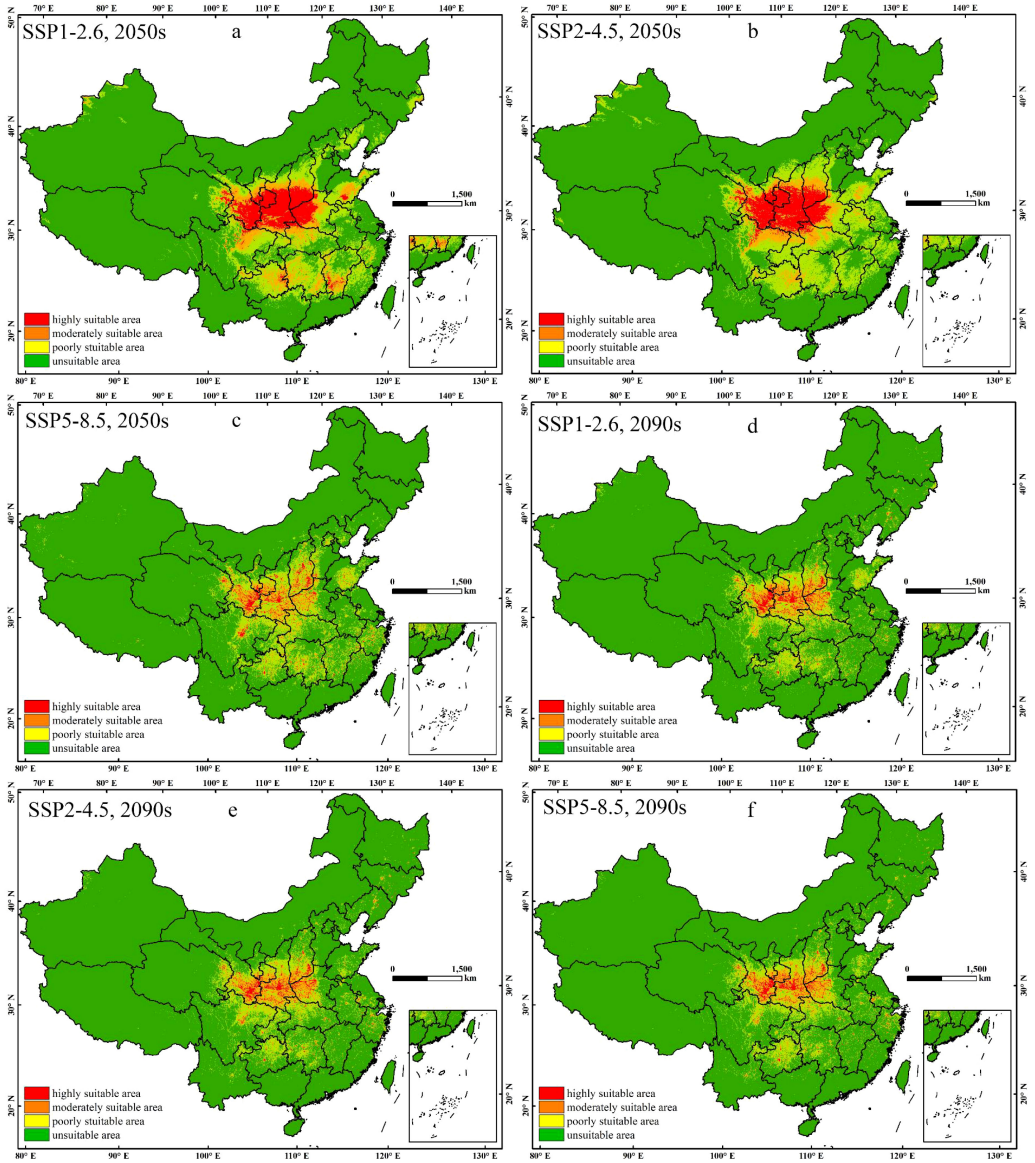
The potential distribution of *E. brevicornu* in suitable regions in China under different climatic conditions. This area’s color blocks display the probability of *E. brevicornu* occurrence. **(a–c)** 2050s under SSP1-2.6, SSP2-4.5, and SSP5-8.5 scenarios; **(d–f)** 2090s under corresponding SSP scenarios. Red indicates a high suitability range of 0.6-1, orange indicates a moderate suitability range of 0.3-0.6, yellow indicates a low suitability range of 0.1-0.3, and green indicates an unsuitable range of 0-0.1.

The suitable range of *E. brevicornu* in the 2050s and 2090s, based on the predictions of the three climate change scenarios SSP1-2.6, SSP2-4.5, and SSP5-8.5, can be seen in **Figure 4**, with all ranges increasing. It is expected that in the 2050s, the area of high suitability for the species would mainly be concentrated in the Loess Plateau and northern Sichuan, with some suitable areas also present in the Yungui Plateau, southern hilly areas, and the North China Plain. Under the SSP1-2.6 scenario, the area of high suitability increased the most, with significant increases in areas such as Guizhou, Hunan, Hubei, Jiangxi, and Shandong. Under the SSP2-4.5 scenario, the area of high suitability in regions such as the Loess Plateau increased compared to SSP1-2.6, but decreased in other areas. The increase in the area of high suitability under the SSP5-8.5 scenario was less pronounced, with unclear differences compared to the current distribution. From the 2050s to the 2090s, the area of high suitability decreased under the SSP1-2.6 and SSP2-4.5 scenarios, approaching the area under the SSP5-8.5 scenario, but still increased compared to the current high suitability area.

According to the predictions, by the 2050s and 2090s, the suitable range for *E. brevicornu* was expected to be wider than present (**Table 4**). In the 2050s, the predicted increase in the suitable range for *E. brevicornu* under the SSP1-2.6, SSP2-4.5, and SSP5-8.5 scenarios was 396.94%, 416.33%, and 17.49%, respectively. Among these, the SSP2-4.5 scenario showed the largest increase in the high suitability range for *E. brevicornu*, followed by the SSP1-2.6 scenario. In the 2090s, the predicted increase in the suitable range for *E. brevicornu* under the SSP1-2.6, SSP2-4.5, and SSP5-8.5 scenarios was 21.06%, 10.61%, and 14.87%, respectively. Compared to the 2050s, the area of high suitability will decrease in the 2090s.

**Table 4:** Suitable areas under current and future climate conditions.

Decade Scenarios		Predicted Area (×10^3^ Km^2^)			Comparison with Current Distribution (%)		
		Poorly suitable aera	Moderately suitable aera	Highly suitable aera	Poorly suitable aera	Moderately suitable aera	Highly suitable aera
Current		72	26	7			
2050s	SSP1-2.6	114.30	43.46	37.24	57.84	64.68	396.94
	SSP2-4.5	126.00	37.53	38.69	74.00	42.22	416.33
	SSP5-8.5	92.72	29.89	8.80	28.05	13.28	17.49
2090s	SSP1-2.6	72.42	28.10	9.07	0.01	6.47	21.06
	SSP2-4.5	73.24	29.54	8.29	1.14	11.94	10.61
	SSP5-8.5	76.01	28.88	8.61	4.97	9.42	14.87

### 3.4 Environmental variables affecting the geographical distribution of *E. brevicornu*

**Figure 5:**
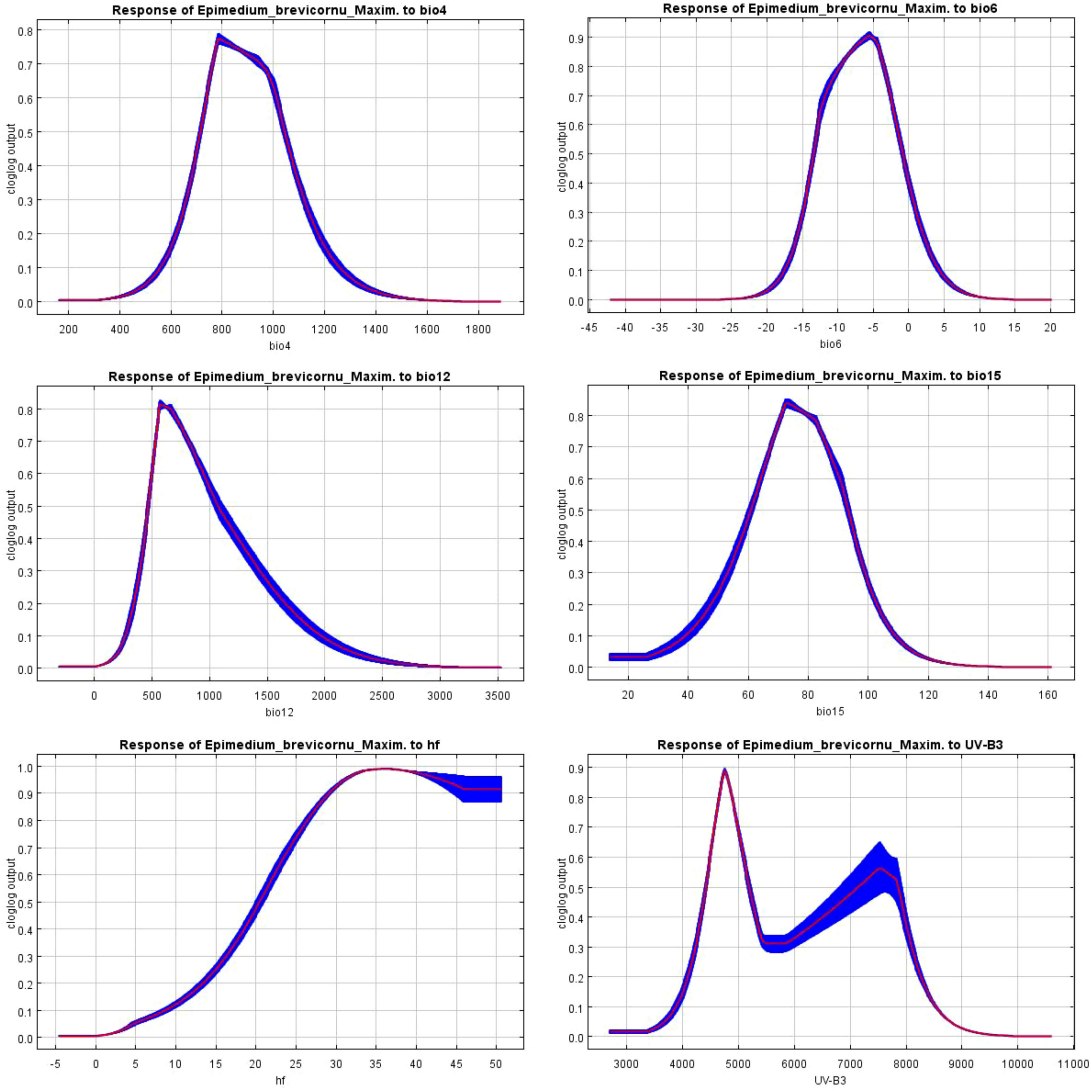
Response curve of *E. brevicornu* to environmental variables.

According to the response curve of *E. brevicornu*’s probability of occurrence in relation to environmental variables in the MaxEnt model (**Figure 5**), the suitable range (optimal value) of environmental variables for *E. brevicornu* distribution was determined (**Table 5**). The seasonal variation coefficient of air temperature ranged from 735.52 to 1013.95°C (mean: 783.64°C), the lowest temperature in the coldest month ranged from -12.88 to -1.67°C (mean: -5.54°C), annual precipitation ranged from 503.96 to 948.71 mm (mean: 582.11 mm), the seasonal variation in precipitation ranged from 64.22 to 90.59 mm (mean: 72.71 mm), human disturbance ranged from 22.31% to 50.00% (mean: 35.44%), and the maximum monthly average UV radiation ranged from 4496.05 to 5111.58 kJ/m² (mean: 4761.76 kJ/m²). Within the suitable range, the probability of occurrence increased as all environmental variable values approached the optimal value, but began to decrease after surpassing the optimum. It was worth noting that the probability of occurrence of *E. brevicornu* showed a secondary increase trend after exceeding 5658.76 kJ/m² for the maximum monthly average UV radiation, although the probability was not very high.

**Table 5:** Potential ranges of suitable environmental variables for *E. brevicornu*.

Environmental variables	Suitable range	Optimal value
bio4(°C)	735.52∼1013.95.43	783.64
bio6(°C)	-12.88∼-1.67	-5.54
bio12(mm)	503.96∼948.71	582.11
bio15(mm)	64.22∼90.59	72.71
Hf(%)	22.31∼50.00	35.44
UV-B3(kJ/m^2^)	4496.05∼5111.58	4761.76

### 3.5 Shift in the centroids of highly suitable habitats under three future climate scenarios

**Figure 6:**
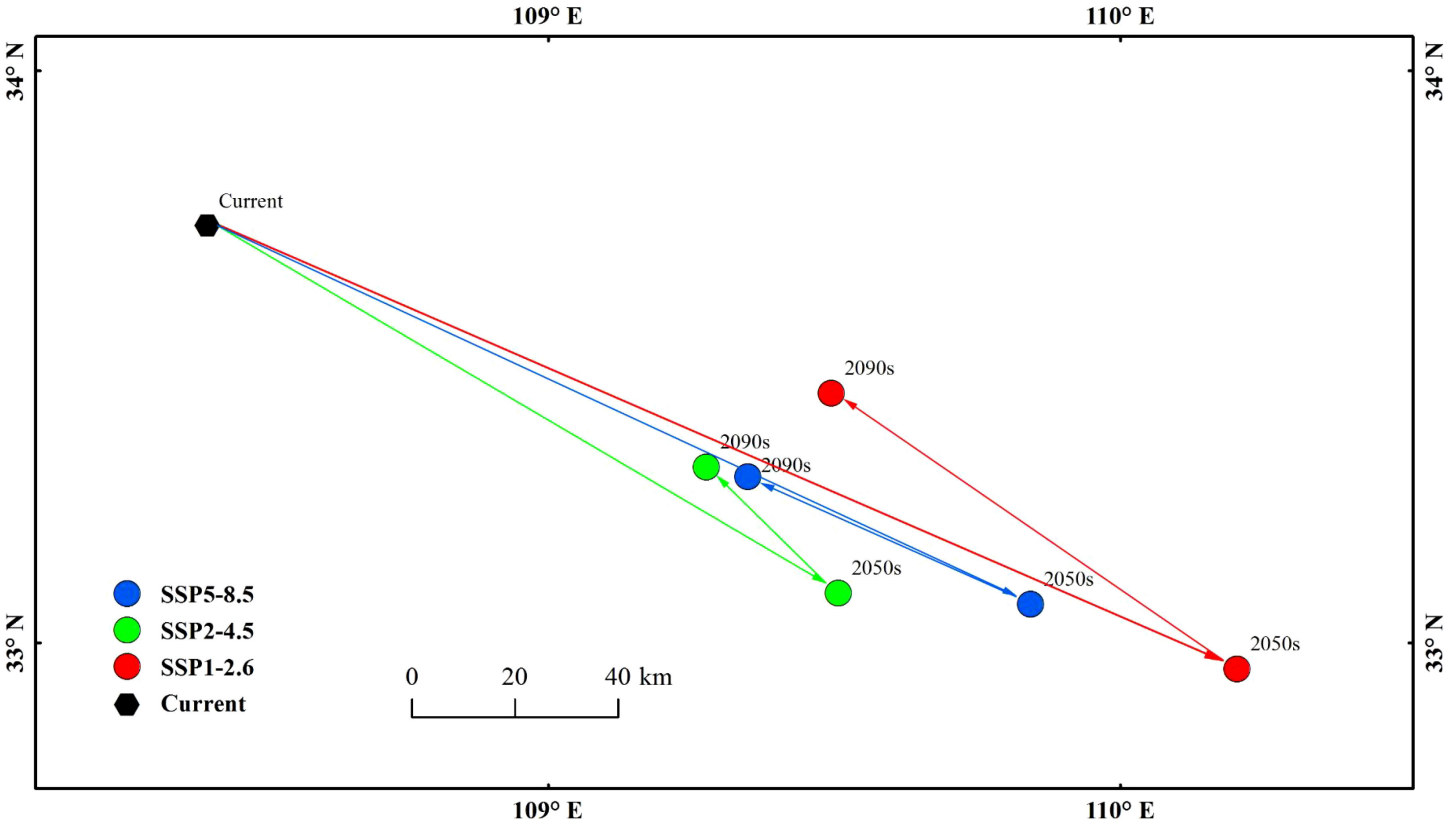
Centroids of highly suitable habitats.

Under the current climatic conditions, the centroid of the high-suitability area for *E. brevicornu* was located in Foping County, Xi’an City, Shaanxi Province (**Figure 6**). In three future climate scenarios, the centroid of the high-suitability area shifted to varying degrees, but the overall direction of the shift was southeast, with the shifted centroids all remaining in Shaanxi Province (**Figure 6**). Under the SSP1-2.6 scenario, by the 2050s, the centroid moved to Yunxi County, Shiyan City (32.95°N, 110.20°E), with a distance of 124.20 km²; by the 2090s, the centroid moved to Zhen’an County, Shangluo City (33.43°N, 109.49°E), with a distance of 112.91 km². Under the SSP2-4.5 scenario, by the 2050s, the centroid moved to Xunyang County, Ankang City (33.08°N, 109.50°E), with a distance of 31.84 km²; by the 2090s, the centroid moved to Zhen’an County, Shangluo City (33.30°N, 109.27°E), with a distance of 96.83 km². Under the SSP5-8.5 scenario, by the 2050s, the centroid moved to Yunxi County, Shiyan City (33.06°N, 109.84°E), with a distance of 54.22 km²; by the 2090s, the centroid moved to Zhen’an County, Shangluo City, Hengyang County (33.28°N, 109.34°E), with a distance of 104.14 km². By the 2050s, the centroids were located in Shiyan City and Ankang City, respectively; by the 2090s, they were both located in Shangluo City. Protected areas for *E. brevicornu* could be established with these cities as the centers.

## 4 Discussion

### 4.1 Predictions and evaluation of MaxEnt model

*Epimedium brevicornu* is widely used as a medicinal plant in both traditional medicine and modern drug preparation. A total of 29 bioclimatic variables were selected as the primary environmental factors, and the MaxEnt model was employed to simulate the potential suitable distribution of *E. brevicornu* in China. According to the results of the MaxEnt model, *E. brevicornu* is currently distributed primarily within the range of 25.13°–39.50°N, 102.46°–118.13°E in China, as well as in the region at 47.29°N, 130.25°E. The most suitable growth areas predominantly include the southern parts of Gansu, Shaanxi, Shanxi, western Henan, northern Sichuan, and northern Hunan provinces in China, which aligns with previous research findings (Ma et al., 2011). This indicates that the Loess Plateau region is more suitable for the survival of *E. brevicornum* compared to other areas. In the future, climate change will have a significant impact on the distribution of *E. brevicornum*. Under the SSP1-2.6 and SSP2-4.5 scenarios, the highly suitable growth areas in the 2050s will expand significantly, primarily in the Loess Plateau, further confirming the species’ preference for distribution in this region. However, by the 2090s, the extent of the highly suitable areas will decrease compared to the 2050s, with the area approaching that under the SSP5-8.5 scenario. In the future, highly suitable areas in all scenarios will increase compared to the current situation.

### 4.2 Key environmental variables and ecological characteristics

The habitat of a species is often closely related to local bioclimatic, topographical, and soil factors (He et al., 2021). In this study, key environmental variables affecting the occurrence probability of *E. brevicornu* were analyzed, and the corresponding response curves were generated. The results demonstrated that the probability of occurrence of *E. brevicornu* varied with changes in key environmental factors. Temperature emerged as the most significant variable affecting its distribution, particularly the seasonal variation coefficient of temperature and the lowest temperature in the coldest month. Precipitation was a secondary factor, with the most significant impacts observed for annual precipitation and seasonal variation in precipitation. In addition, human footprint and UV-B radiation were also found to influence the distribution of *E. brevicornu*.

Temperature plays a dominant role in plant growth and development (Gao et al., 2022) and influences plant geographical distribution (Ding and Yang, 2022). Studies have shown that differences in species’ global distribution are largely determined by their cold tolerance levels (Araújo et al., 2013). In this study, *E. brevicornum* was found to be most suitable at a minimum temperature of -5.38°C, where the probability of occurrence was highest. Low temperatures can affect seed dormancy and germination, thus influencing the plant’s growth cycle (Inouye, 2020). *E. brevicornum* seeds exhibit dormancy, and the optimal temperature range for dormancy is 2-6°C, which is consistent with the modeling results (Li et al., 2024a). This suggests that the temperature limitation for *E. brevicornum* is primarily during the seed dormancy period. Furthermore, some studies have shown that the growth of *E. brevicornum* increases significantly with a 2°C temperature rise compared to a 5°C rise (Xuemei et al., 2016). This indicates that *E. brevicornum* is better suited to moderate temperature changes and struggles to adapt to more extreme temperature shifts. The temperature rise is higher under the SSP5-8.5 scenario, while it is smaller under the SSP1-2.6 and SSP2-4.6 scenarios. As a result, the highly suitable areas in future low-emission scenarios will increase more significantly, while the changes in high-emission scenarios will be smaller. Precipitation changes can affect species distribution and have been shown to maintain relationships between different plant species (Ding and Yang, 2022). When precipitation increases excessively, the productivity of perennial herbaceous plants decreases (Gherardi and Sala, 2015), likely because excess moisture causes root rot and promotes the growth of pests and diseases (Duan et al., 2022). The most suitable conditions for *E. brevicornum* growth occur when the annual precipitation is 582.11 mm and the seasonal precipitation variation is 72.36 mm, which is similar to the precipitation levels in the Loess Plateau (Wu et al., 2024). Research has found that natural drought in the soil after rainfall during the harvest period can enhance the quality of related plants (Zhang et al., 2024b), which could explain why *E. brevicornum* is widely distributed in the Loess Plateau. Human footprint can alter terrestrial ecosystems, and the extent and magnitude of these changes vary over time and space (Watson and Venter, 2019), with environmental changes caused by human activity being complex and diverse (Kennedy et al., 2019). Although the inclusion of human footprint in SDM models is relatively recent, human activity remains one of the most important factors influencing plant distribution (Amat et al., 2013; Gallardo et al., 2015) and has different effects on various plants (Bai et al., 2024), promoting the distribution of some high-altitude plants (Amat et al., 2013). In this study, it was found that as the human footprint increased, the probability of *E. brevicornum* distribution also increased. Although this probability decreased after reaching 35.44%, it remained relatively high, suggesting that human activity may promote the distribution of *E. brevicornum*. UV-B radiation can cause changes in plant metabolism, physiology, and development (Liu et al., 2024). UV-B stress reduces the leaf area of *E. brevicornum* but increases the accumulation of flavonoid compounds (Li et al., 2024b). The model indicated that when the highest monthly UV-B radiation ranged between 4587.88 and 4769.53 kJ/m², it had a positive impact. However, as UV-B radiation exceeded 4769.53 kJ/m² and approached 5658.76 kJ/m², the probability of occurrence decreased with increasing UV-B radiation. When the radiation exceeded 5658.76 kJ/m², the occurrence probability showed a secondary increase, although the peak probability was lower. This may be because plants adapt to UV-B stress to protect themselves (Shi and Liu, 2021). This has been confirmed in other high-altitude plant species (Shi et al., 2022), but further research is needed for *E. brevicornum*, which is widely distributed in the Loess Plateau.

### 4.3 The protection strategy of short-horned rye grass

The wild populations of *E. brevicornum* are under threat, making conservation efforts essential. The primary task in conserving *E. brevicornum* should be protecting its native ecological environment. Based on modeling results, conservation efforts should focus on the main distribution area of *E. brevicornum*, the Loess Plateau, taking into full consideration the key environmental factors that influence its growth. Secondly, expanding artificial cultivation is important. At present, the cultivation of medicinal plants by replicating the living conditions of wild plants has already been implemented (He et al., 2021). Studies have shown that the centers of high suitability areas for *E. brevicornum* are in Shangluo, Shiyan, and Ankang cities, as well as their surrounding regions. Therefore, efforts should be focused on conducting surveys, collecting resources, and protecting these areas, which should also be promoted as important bases for planting and development. Finally, human excavation activities should be restricted to protect *E. brevicornum*. Frequent human activities can limit the ability of protected areas to maintain ecosystem integrity (Xia et al., 2021). As a medicinal plant, *E. brevicornum* is subject to exploitation of its wild resources, which threatens its wild populations. Remote sensing can monitor human activities and facilitates the establishment and assessment of protected areas (Xia et al., 2024). Therefore, in the future wild conservation areas of *E. brevicornum*, remote sensing and other geospatial technologies could be integrated in practical applications to monitor and restrict human activities, which would be more beneficial for the conservation of wild *E. brevicornum* resources.

There were still some limitations in the model prediction process. First, the role of the model was limited to prediction, and the extent of future environmental changes may differ from the three scenarios used in the model (Meinshausen et al., 2022). Moreover, rising temperatures are expected to cause extreme climatic events in the Loess Plateau region (Ta et al., 2022). Since not all environmental variables could be taken into account (Xu et al., 2022), prediction errors are inevitable, and the future distribution of *E. brevicornu* may also differ from the predicted patterns. In addition, interspecific interactions, such as competition, predation, and mutualisms, were not fully considered (Van Ee et al., 2022). These factors were not included in the modeling and were not thoroughly investigated in this study, but they may potentially affect the distribution of *E. brevicornum*. Therefore, in practical applications, the model should be used only as a reference. After identifying suitable habitats, effective conservation of the species requires integrating more local environmental factors.

## 5 Conclusion

The MaxEnt model was used to study the geographical distribution and environmental suitability of *Epimedium brevicornu*. The results indicate that, under three different climate change scenarios, the low, medium, and high suitability ranges for *E. brevicornu* all expand in the future, with the centroid of the high suitability range shifting southeast compared to the current distribution. The increase in the high suitability range is particularly notable in the 2050s under the SSP1-2.6 and SSP2-4.5 scenarios. The current suitable growth range of E. brevicornum spans from 25.13° - 39.50°N and from 102.46° - 118.13°E. Key biotic environmental factors, including the Temperature Seasonality (bio4), the Min Temperature of Coldest Month (bio6), annual precipitation (bio12), precipitation seasonality (bio15), human footprint index (HF), and Mean UV-B of Highest Month (UV-B3), significantly influence the distribution of *E. brevicornu*. This study provides predictions of the potential distribution of *E. brevicornu*, which can serve as a valuable reference for the conservation of wild populations and the artificial cultivation of this species.

## Data Availability

The data supporting the results are available in a public repository at: https://figshare.com/s/a509750b43d1e0153d42.
