# Supplementary material for: Assessing the climate change impact on Epimedium brevicornu in China with the MaxEnt model
Source: Front Plant Sci. 2025 Jun 16;16:1534608. doi: 10.3389/fpls.2025.1534608 (PMC12206714; doi:10.3389/fpls.2025.1534608)
Supplement: Supplementary file 1 [file Table1.docx]

Table s1 List of Environmental Variables Used in the Modeling Process

| Code | Environmental variables | Unit |
| --- | --- | --- |
| bio1 | Annual Mean Temperature | ℃ |
| Bio2 | Mean Diurnal Range | ℃ |
| Bio3 | Isothermality | % |
| Bio4 | Temperature Seasonality | ℃ |
| Bio5 | Max Temperature of Warmest Month | ℃ |
| Bio6 | Min Temperature of Coldest Month | ℃ |
| Bio7 | Temperature Annual Range | ℃ |
| Bio8 | Mean Temperature of Wettest Quarter | ℃ |
| Bio9 | Mean Temperature of Driest Quarter | ℃ |
| bio10 | Mean Temperature of Warmest Quarter | ℃ |
| bio11 | Mean Temperature of Coldest Quarter | ℃ |
| bio12 | Annual Precipitation | mm |
| bio13 | Precipitation of Wettest Month | mm |
| bio14 | Precipitation of Driest Month | mm |
| bio15 | Precipitation Seasonality | mm |
| bio16 | Precipitation of Wettest Quarter | mm |
| bio17 | Precipitation of Driest Quarter | mm |
| bio18 | Precipitation of Warmest Quarter | mm |
| bio19 | Precipitation of Coldest Quarter | mm |
