# Supplementary material for: Assessing the climate change impact on Epimedium brevicornu in China with the MaxEnt model
Source: Front Plant Sci. 2025 Jun 16;16:1534608. doi: 10.3389/fpls.2025.1534608 (PMC12206714; doi:10.3389/fpls.2025.1534608)
Supplement: Supplementary file 2 [file Table2.docx]

Table S2 Percentage Contribution and Ranking of Environmental Variables in MaxEnt Model

| Variable | Percent contribution（%） | Permutation importance |
| --- | --- | --- |
| bio6 | 29.1 | 10.8 |
| bio12 | 17.2 | 22.3 |
| bio15 | 14.3 | 5.4 |
| bio14 | 9.5 | 1.8 |
| bio4 | 9.4 | 5.1 |
| bio19 | 7 | 7.1 |
| bio18 | 6.1 | 2 |
| bio3 | 3.2 | 1.6 |
| bio8 | 1.5 | 17.5 |
| bio11 | 1.1 | 0 |
| bio10 | 0.9 | 25.3 |
| bio5 | 0.5 | 0 |
| bio13 | 0.2 | 0.2 |
| bio17 | 0.1 | 0.5 |
| bio1 | 0.1 | 0.2 |
| bio1 | 0.1 | 0.2 |
| bio2 | 0 | 0.1 |
| bio7 | 0 | 0 |
| bio9 | 0 | 0 |
