# Supplementary material for: Assessing the climate change impact on Epimedium brevicornu in China with the MaxEnt model
Source: Front Plant Sci. 2025 Jun 16;16:1534608. doi: 10.3389/fpls.2025.1534608 (PMC12206714; doi:10.3389/fpls.2025.1534608)
Supplement: Supplementary file 3 [file Table3.docx]

Table s3 correlation analysis

|  | bio1 | bio3 | bio4 | bio5 | bio6 | bio8 | bio10 | bio11 | bio12 | bio13 | bio14 | bio15 | bio17 | bio18 | bio19 |
| --- | --- | --- | --- | --- | --- | --- | --- | --- | --- | --- | --- | --- | --- | --- | --- |
| bio1 | 1.00 |  |  |  |  |  |  |  |  |  |  |  |  |  |  |
| bio3 | -0.51^**^ | 1.00 |  |  |  |  |  |  |  |  |  |  |  |  |  |
| bio4 | -0.08 | -0.41^**^ | 1.00 |  |  |  |  |  |  |  |  |  |  |  |  |
| bio5 | 0.89^**^ | -0.56^**^ | 0.35^**^ | 1.00 |  |  |  |  |  |  |  |  |  |  |  |
| bio6 | 0.90^**^ | -0.34^**^ | -0.49^**^ | 0.62^**^ |  |  |  |  |  |  |  |  |  |  |  |
| bio8 | 0.90^**^ | -0.55^**^ | 0.27^**^ | 0.94^**^ | 0.66^**^ | 1.00 |  |  |  |  |  |  |  |  |  |
| bio10 | 0.94^**^ | -0.63^**^ | 0.25^*^ | 0.98^**^ | 0.71^**^ | 0.96^**^ | 1.00 |  |  |  |  |  |  |  |  |
| bio11 | 0.93^**^ | -0.32^**^ | -0.43^**^ | 0.68^**^ | 0.96*^**^ | 0.70^**^ | 0.76^**^ |  |  |  |  |  |  |  |  |
| bio12 | 0.63^**^ | -0.42^**^ | -0.40^**^ | 0.39^**^ | 0.74^**^ | 0.33^**^ | 0.46^**^ | 0.72^**^ | 1.00 |  |  |  |  |  |  |
| bio13 | 0.61^**^ | -0.40^**^ | -0.27^**^ | 0.43^**^ | 0.65^**^ | 0.40^**^ | 0.47^**^ | 0.64^**^ | 0.92^**^ | 1.00 |  |  |  |  |  |
| bio14 | 0.64^**^ | -0.52^**^ | -0.20 | 0.51^**^ | 0.64^**^ | 0.36^**^ | 0.54^**^ | 0.65^**^ | 0.91^**^ | 0.77^**^ | 1.00 |  |  |  |  |
| bio15 | -0.60^**^ | 0.41^**^ | 0.36^**^ | -0.39^**^ | -0.72^**^ | -0.35^**^ | -0.47^**^ | -0.69^**^ | -0.69^**^ | -0.40^**^ | -0.72^**^ | 1.00 |  |  |  |
| bio17 | 0.61^**^ | -0.51^**^ | -0.20 | 0.48^**^ | 0.63^**^ | 0.33^**^ | 0.51^**^ | 0.63^**^ | 0.91^**^ | 0.76^**^ | 1.00 ^**^ | -0.72^**^ | 1.00 |  |  |
| bio18 | 0.51^**^ | -0.32^**^ | -0.34^**^ | 0.29^**^ | 0.59^**^ | 0.30^**^ | 0.35^**^ | 0.57^**^ | 0.89^**^ | 0.98^**^ | 0.69^**^ | -0.33^**^ | 0.68^**^ | 1.00 |  |
| bio19 | 0.59^**^ | -0.48^**^ | -0.19 | 0.47^**^ | 0.61^**^ | 0.30^**^ | 0.49^**^ | 0.62^**^ | 0.89^**^ | 0.74^**^ | 0.99^**^ | -0.70^**^ | 0.99^**^ | 0.65^**^ | 1.00 |
