# Supplementary material for: Assessing the climate change impact on Epimedium brevicornu in China with the MaxEnt model
Source: Front Plant Sci. 2025 Jun 16;16:1534608. doi: 10.3389/fpls.2025.1534608 (PMC12206714; doi:10.3389/fpls.2025.1534608)
Supplement: Supplementary file 4 [file Table4.docx]

Table S4 Predicted area of China pepper under current climate conditions

| Province | Unsuitable Aera | | Poorly  Suitable  Aera | | Moderately  Suitable  Aera | | Highly  Suitable  Aera | |
| --- | --- | --- | --- | --- | --- | --- | --- | --- |
|  | Predicted area（×10^3^ km^2^ ) | Area ratio  (%) | Predicted area （×10^3^ km^2^ ) | Area ratio  (%) | Predicted area （×10^3^ km^2^ ) | Area ratio  (%) | Predicted area （×10^3^ km^2^ ) | Area ratio  (%) |
| Heilongjiang | 539.55 | 99.33 | 2.71 | 0.50 | 0.90 | 0.17 | 0.05 | 0.01 |
| Xinjiang | 1712.71 | 97.52 | 40.24 | 2.29 | 3.04 | 0.17 | 0.23 | 0.01 |
| Jilin | 205.00 | 96.29 | 6.11 | 2.87 | 1.18 | 0.55 | 0.61 | 0.29 |
| Liaoning | 148.78 | 95.41 | 6.02 | 3.86 | 0.99 | 0.63 | 0.14 | 0.09 |
| Gansu | 265.09 | 63.83 | 70.61 | 17.00 | 62.48 | 15.05 | 17.12 | 4.12 |
| Hebei | 186.15 | 94.85 | 8.73 | 4.45 | 1.22 | 0.62 | 0.16 | 0.08 |
| Shanxi | 66.65 | 41.77 | 52.53 | 32.92 | 29.11 | 18.25 | 11.27 | 7.06 |
| Shaanxi | 36.02 | 17.67 | 79.06 | 38.79 | 64.79 | 31.79 | 23.94 | 11.75 |
| Ningxia | 35.10 | 66.64 | 11.02 | 20.93 | 5.71 | 10.84 | 0.83 | 1.58 |
| Qinghai | 680.30 | 95.36 | 26.37 | 3.70 | 5.09 | 0.71 | 1.65 | 0.23 |
| Shandong | 122.20 | 79.68 | 28.42 | 18.53 | 2.66 | 1.73 | 0.09 | 0.06 |
| Henan | 72.45 | 44.92 | 47.48 | 29.44 | 33.54 | 20.80 | 7.81 | 4.84 |
| Anhui | 119.65 | 89.54 | 12.24 | 9.16 | 1.39 | 1.04 | 0.35 | 0.26 |
| Sichuan | 349.46 | 76.75 | 90.75 | 19.93 | 12.83 | 2.82 | 2.31 | 0.51 |
| Hubei | 110.14 | 62.72 | 54.29 | 30.91 | 9.77 | 5.57 | 1.41 | 0.80 |
| Chongqing | 48.45 | 62.63 | 25.38 | 32.81 | 3.13 | 4.04 | 0.40 | 0.52 |
| Zhejiang | 72.31 | 77.84 | 15.16 | 16.31 | 4.13 | 4.45 | 1.30 | 1.40 |
| Hunan | 136.32 | 70.31 | 46.72 | 24.10 | 8.82 | 4.55 | 2.03 | 1.05 |
| Jiangxi | 132.14 | 86.53 | 16.51 | 10.81 | 3.11 | 2.04 | 0.95 | 0.63 |
| Yunnan | 328.02 | 95.69 | 13.11 | 3.82 | 1.60 | 0.47 | 0.09 | 0.03 |
| Guizhou | 105.00 | 65.78 | 47.62 | 29.83 | 5.45 | 3.42 | 1.55 | 0.97 |
| Fujian | 101.67 | 94.01 | 4.77 | 4.41 | 1.25 | 1.16 | 0.45 | 0.42 |
| Guangxi | 204.93 | 97.97 | 3.59 | 1.72 | 0.54 | 0.26 | 0.10 | 0.05 |
| Guangdong | 150.45 | 98.45 | 1.98 | 1.30 | 0.28 | 0.18 | 0.10 | 0.07 |
